# High Levels of Antibiotic Resistance Genes and Their Correlations with Bacterial Community and Mobile Genetic Elements in Pharmaceutical Wastewater Treatment Bioreactors

**DOI:** 10.1371/journal.pone.0156854

**Published:** 2016-06-13

**Authors:** Wenda Tao, Xu-Xiang Zhang, Fuzheng Zhao, Kailong Huang, Haijun Ma, Zhu Wang, Lin Ye, Hongqiang Ren

**Affiliations:** State Key Laboratory of Pollution Control and Resource Reuse, Environmental Health Research Center, School of the Environment, Nanjing University, Nanjing, 210023, China; Nankai University, CHINA

## Abstract

To understand the diversity and abundance of antibiotic resistance genes (ARGs) in pharmaceutical wastewater treatment bioreactors, the ARGs in sludge from two full-scale pharmaceutical wastewater treatment plants (PWWTPs) were investigated and compared with sludge samples from three sewage treatment plants (STPs) using metagenomic approach. The results showed that the ARG abundances in PWWTP sludge ranged from 54.7 to 585.0 ppm, which were higher than those in STP sludge (27.2 to 86.4 ppm). Moreover, the diversity of ARGs in PWWTP aerobic sludge (153 subtypes) was higher than that in STP aerobic sludge (118 subtypes). In addition, it was found that the profiles of ARGs in PWWTP aerobic sludge were similar to those in STP aerobic sludge but different from those in PWWTP anaerobic sludge, suggesting that dissolve oxygen (DO) could be one of the important factors affecting the profiles of ARGs. In PWWTP aerobic sludge, aminoglycoside, sulfonamide and multidrug resistance genes were frequently detected. While, tetracycline, macrolide-lincosamide-streptogramin and polypeptide resistance genes were abundantly present in PWWTP anaerobic sludge. Furthermore, we investigated the microbial community and the correlation between microbial community and ARGs in PWWTP sludge. And, significant correlations between ARG types and seven bacterial genera were found. In addition, the mobile genetic elements (MGEs) were also examined and correlations between the ARGs and MGEs in PWWTP sludge were observed. Collectively, our results suggested that the microbial community and MGEs, which could be affected by DO, might be the main factors shaping the profiles of ARGs in PWWTP sludge.

## Introduction

As potential threats to human health, antibiotic resistant bacteria (ARB) and antibiotic resistance genes (ARGs) continue to spread globally due to the overuse and misuse of antibiotics for medical treatment, veterinary and agriculture [1–3]. In recent years, ARGs have been detected in various environments, such as soil [4], groundwater [5], sediment [6], etc. Many studies have showed that wastewater treatment plants (WWTPs) are significant sources of ARGs in the natural environment [7–9]. Moreover, previous studies also reported that the discharge of effluent and sludge was one of the main routes to release the ARGs to the environment and the dissemination of ARGs to the environment from sludge was about 1000 times higher than effluent [10].

According to a study conducted by Gao et al. [11], positive correlations were found between some ARGs and their corresponding antibiotics in a sewage treatment plant (STP). Therefore, pharmaceutical wastewater treatment plants (PWWTPs) are worthy of attention due to the high concentration of antibiotics in the pharmaceutical wastewater [12]. As a vital node in PWWTPs, biological treatment process with a high density of bacteria created an ideal environment for ARG exchange [13] through horizontal gene transfer (HGT) among different microorganisms, which was controlled by mobile genetic elements (MGEs), including plasmids, integrons and insertion sequences (ISs) [14–16]. Moreover, the HGT may cause ARGs to be transferred to pathogenic bacteria, which could pose serious health risks to humans [17, 18].

A few studies have been conducted to investigate the ARGs in PWWTPs [19, 20]. However, the pioneer studies provided limited information of ARGs due to the limitations of the quantitative polymerase chain reaction (q-PCR) methodology. Metagenomic approaches could overcome the drawbacks of amplification-based methods and have been successfully applied to investigate ARGs in various environmental samples, such as, soil [21], biofilm [22], deep ocean sediments [23], wastewater and activated sludge [24].

The present study investigated the abundance and diversity of ARGs, MGEs and bacterial community in activated sludge samples from PWWTPs and STPs by using metagenomic sequencing. The objectives of this study were (1) to explore the abundance and diversity of ARGs and MGEs in PWWTP sludge (2) to reveal the potential differences of ARGs between anaerobic and aerobic treatment processes (3) to investigate the potential reasons on the variation of ARGs among different treatment processes. The findings of this study may help to extend our knowledge about the distribution of ARGs and the correlations between ARGs and bacterial community and MGEs in PWWTP sludge.

## Materials and Methods

### Sampling and DNA extraction

**Fig 1:**
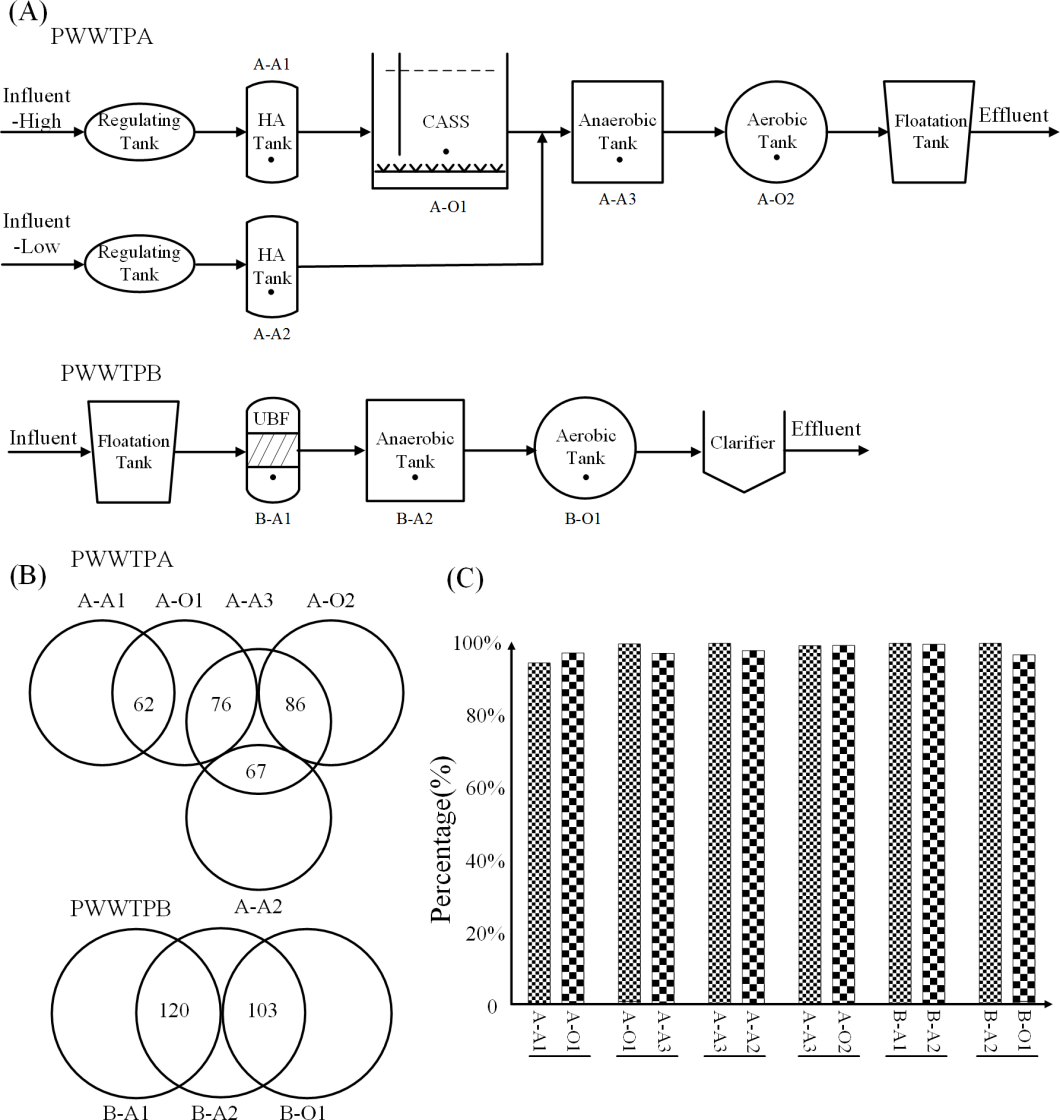
Treatment processes in the two PWWTPs and the shared ARGs. (A) The flow charts and sampling sites of the treatment processes in two PWWTPs. The black points in oxidation ditch represent the sampling sites. HA: Hydrolytic Acidification; CASS: Cyclic Activated Sludge System; UBF: Up-flow Blanket Filter; (B) Number of shared ARG subtypes by adjacent treatment systems in two PWWTPs. (C) Percentage of the shared ARGs in the total ARGs.

In this study, eleven sludge samples were collected from two full-scale PWWTPs of Hisun Pharmaceutical Co., Ltd located in two cities (PWWTP A in Hangzhou City and PWWTP B in Taizhou City, Zhejiang Province) of China and three full-scale STPs located in Zhengzhou, Henan Province, China, and, we would like to state that these plants have approved this study. Relevant operational parameters about the PWWTPs and STPs were shown in S1 Table. The flowcharts and the sampling sites of the two PWWTPs were shown in Fig 1A. PWWTP A was composed of two hydrolytic acidification systems (HA), a cyclic activated sludge system (CASS) and an anaerobic/oxic system (A/O). The process of up-flow blanket filter system (UBF) and anaerobic/oxic system (A/O) were applied in PWWTP B. The flowcharts and the sampling sites of the three STPs were shown in S1 Fig. The process of anaerobic/anoxic/oxic system (A/A/O) was applied in STP-1 and STP-2 and the process of oxidation ditch (OD) was applied in STP-3. Eight sludge samples were collected from PWWTPs, three sludge samples were collected from STPs and the sample IDs were shown in S1 Table. The sludge samples were immediately mixed with 50% ethanol (v/v) before transporting to our laboratory for DNA extraction. The fixed sludge was centrifuged at 4,000 rpm for 10 min to collect approximately 200 mg of the pellets for total genomic DNA extraction with the FastDNA^®^ Spin kit for Soil (MP Biomedicals, CA, USA) [25]. The concentration and quality of the extracted DNA were determined with microspectrophotometry (NanoDrop ND-2000, NanoDrop Technologies, Willmington, DE, USA).

### Illumina high-throughput sequencing and quality filtering

The DNA samples extracted from activated sludge were sent to Jiangsu Zhongyijinda Analytical & Testing Co., Ltd for library construction and high-throughput sequencing on a Hiseq2500 platform (Illumina, San Diego, CA, USA). The sequencing strategy of Index 101 PE (Paired-End sequencing, 101-bp reads and 8-bp index sequence) was applied to generate raw sequencing reads. For quality control, the raw sequences contaminated by adapter or containing three or more unknown nucleotides (‘N’) were firstly removed using the quality control (QC) pipeline recommended by sequencing institutions. A strict filtration strategy was then conducted by using Galaxy (https://usegalaxy.org/). ‘FASTQ Groomer’ tool was used to convert quality formats, and ‘Filter by quality’ tool was then used to remove low quality sequences to ensure that more than 75% bases of each filtered read with quality greater than 30. The quality-filtered reads were used for subsequent analysis. The metagenomic sequencing data have been deposited into sequence read archive (SRA) database under accession number PRJNA 316198.

### Bioinformatics Analysis

In order to evaluate the distribution of ARGs and MGEs in PWWTPs and STP sludge, the quality-filtered reads were separately aligned to databases including Antibiotic Resistance Genes Database (ARDB, http://ardb.cbcb.umd.edu/index.html) [26], The Integron Database (INTEGRALL, http://integrall.bio.ua.pt/) [27], Insertion Sequences Database (IS Finder, https://www-is.biotoul.fr//) [28] and NCBI Reference Sequence Database (NCBI RefSeq database, http://www.ncbi.nlm.nih.gov/refseq) [29]. A read was annotated as ARG sequences according to its best BLAST hit in ARDB with a threshold of amino acid sequence identity≥90% and sequence alignment length≥25 amino acids [30]. A read was identified as integron or insertion sequence if the sequence had an identity ≥90% with its best BLAST (BLASTn with the E-value cut-off at 10^−5^) hit over an alignment of at least 50 bp [30]. The plasmids identification was determined by using BLAST (BLASTn with the E-value cut-off at 10^−5^) with a threshold of nucleotide sequence identity ≥95% over an alignment of at least 90 bp [30].

The quality-filtered Illumina reads of the eight sludge samples were submitted to Metagenomics RAST server (MG-RAST) (http://metagenomics.anl.gov/) [31]. The MG-RAST server was used to compare the reads of the PWWTP sludge samples using ‘best hit classification’ function with Ribosomal Database Project (RDP) database as the annotation source with a maximum Evalue cutoff of 10^−5^, a minimum identity of 97%, and a minimum alignment length of 50 bp [32, 33].

### Statistical Analysis

In order to analyze the abundance of ARGs and MGEs in different samples, the portion of ARGs and MGEs in “total metagenome sequences” was defined as “abundance” (using the unit of “ppm”, one read in one million reads). The portion of ARGs and 16S rRNA gene sequences in “total ARG sequences” and “total 16S rRNA gene sequences” were defined as “relative abundance” (expressed as percentage to avoid confusion). Redundancy analysis (RDA) was performed by using ‘vegan’ package (version 2.0–10) of R software (version 3.1.0). Principal Coordinates Analysis (PCoA) and the Pearson correlation analysis were performed by using PAleontological STatistics software (PAST, version 3.01). Linear regression analysis of the total ARGs and MGEs was carried out by using Statistical Product and Service Solutions software (SPSS, version 22.0). Student’s *t* test was carried out to assess the variations and *p* < 0.05 was considered to be statistically significant.

## Results

### Diversity of ARGs in PWWTP and STP sludge

In this study, 215 ARG subtypes belonging to 11 ARG types (aminoglycoside, beta-lactam, chloramphenicol, fosfomycin, macrolide-lincosamide-streptogramin (M-L-S), multidrug, polypeptide, quinolone, sulfonamide, tetracycline and unclassified) were identified from the 11 sludge samples by high-throughput sequencing-based metagenomic approach (S2 Fig). In general, three ARG types (fosfomycin, quinolone and unclassified) were present in very low abundance (<1%) in all samples. In order to simplify the results, these ARG types were considered as “others” for subsequent analysis. Among all ARG types, multidrug had the highest diversity (39 subtypes), followed by polypeptide (33 subtypes), beta-lactam (31 subtypes), tetracycline (26 subtypes), M-L-S (22 subtypes), aminoglycoside (22 subtypes), chloramphenicol (18 subtypes) and sulfonamide (15 subtypes).

As shown in Fig 1B, most of the ARG subtypes in PWWTPs were shared by the adjacent treatment systems. For instance, between the 151 ARG subtypes of B-A1 and the 132 ARG subtypes of B-A2, there were 120 shared ARG subtypes. Moreover, as shown in Figure A in S1 File, the diversity of ARG subtypes in PWWTP aerobic sludge (153 subtypes) was obviously higher than that in STP aerobic sludge (118 subtypes) and 94 ARG subtypes were shared between these two kinds of sludge samples. Furthermore, these shared ARG subtypes represented over 90% of the total ARG abundance as shown in Fig 1C and Figure B in S1 File. These results implied that the sludge flow may play a critical role in the dissemination of ARGs in PWWTPs and the dominated ARG subtypes in PWWTPs and STP aerobic sludge are largely similar.

### Abundance of ARGs in PWWTP and STP sludge

**Fig 2:**
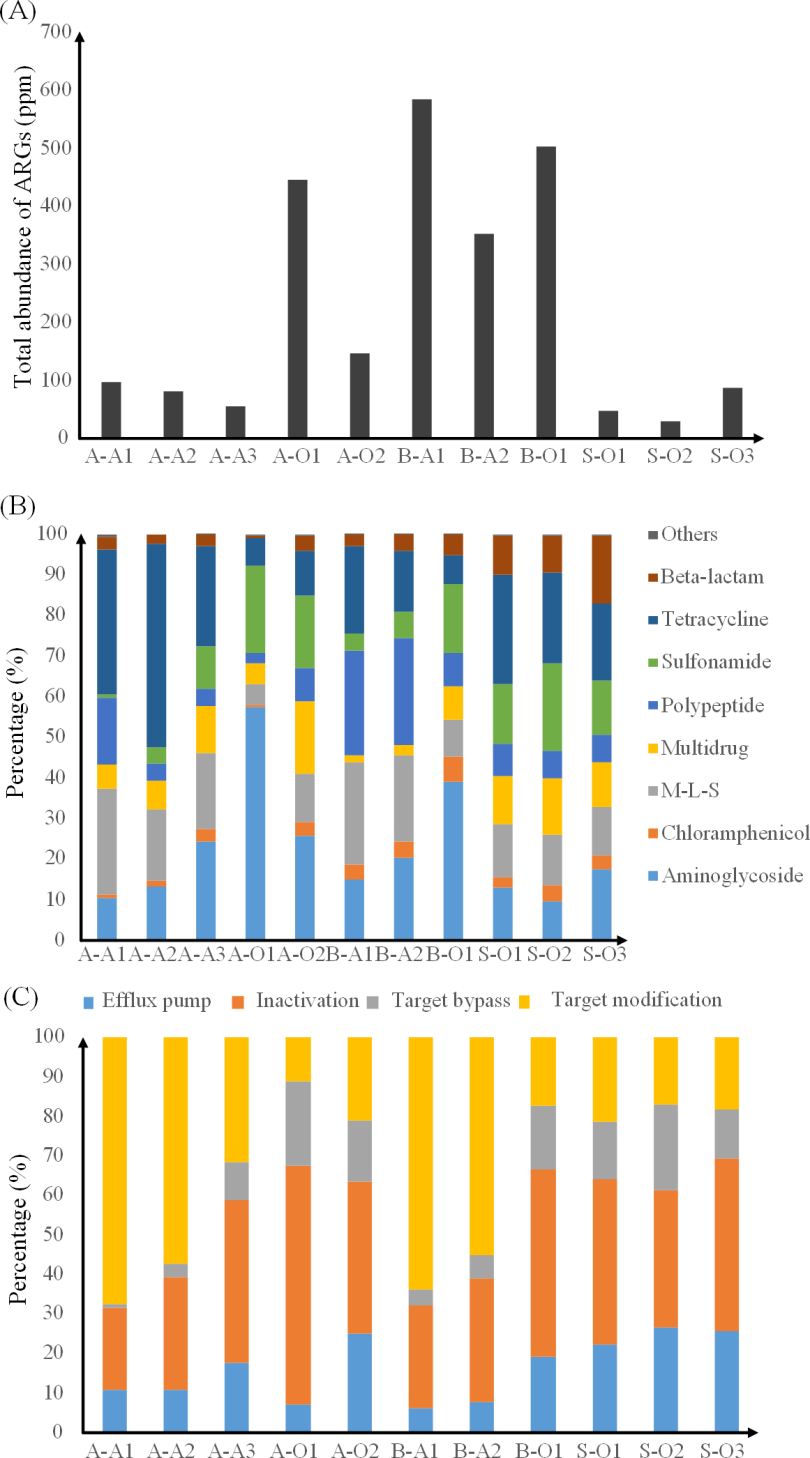
Abundance of ARGs in the PWWTP and STP sludge samples. (A) Total abundance of ARGs in the PWWTP and STP sludge. (B) Percentage of the ARG types in the PWWTP and STP sludge. (C) Percentage of resistance mechanisms in the PWWTP and STP sludge.

In addition to the diversity, it was found that the ARG abundance in PWWTPs was also obviously higher than that in STPs. The total ARG abundance in PWWTPs ranged from 54.7 to 585.0 ppm and the total ARG abundance in STPs ranged from 27.2 to 86.4 ppm (Fig 2A). A summary of the percentage of different ARG types in PWWTPs and STP sludge was shown in Fig 2B and S3 Fig. It was found that the distributions of ARG types in the three STP sludge were similar, tetracycline type had the highest percentage (average 22.8%) and chloramphenicol type had the lowest percentage (average 3.3%). By contrast, the distributions of ARG types in the eight PWWTP sludge samples were different. The percentages of tetracycline, polypeptide and M-L-S in PWWTP anaerobic sludge (average 29.5%, 15.5% and 21.8%, respectively) were much higher than those in PWWTP aerobic sludge (average 8.4%, 6.3% and 8.7%, respectively). While, the percentages of aminoglycoside, sulfonamide and multidrug in PWWTP anaerobic sludge (average 16.5%, 5.2% and 5.8%, respectively) were lower than those in PWWTP aerobic sludge (average 40.6%, 18.8% and 10.4%, respectively).

Fig 2C and S4 Fig showed the percentages of resistance mechanisms in PWWTP and STP sludge. Inactivation was the predominant resistance mechanism (average 40.2%) in STP sludge followed by efflux pump (average 24.7%). Different from the STP sludge, target modification (average 55.2%) was the predominant resistance mechanism in PWWTP anaerobic sludge, followed by inactivation (average 29.8%). While, inactivation (average 48.9%) was the predominant resistance mechanism in PWWTP aerobic sludge, followed by target bypass (average 17.7%). Moreover, the percentage of target modification in PWWTP anaerobic sludge (average 55.2%) was higher than that in PWWTP aerobic sludge (average 16.5%).

**Fig 3:**
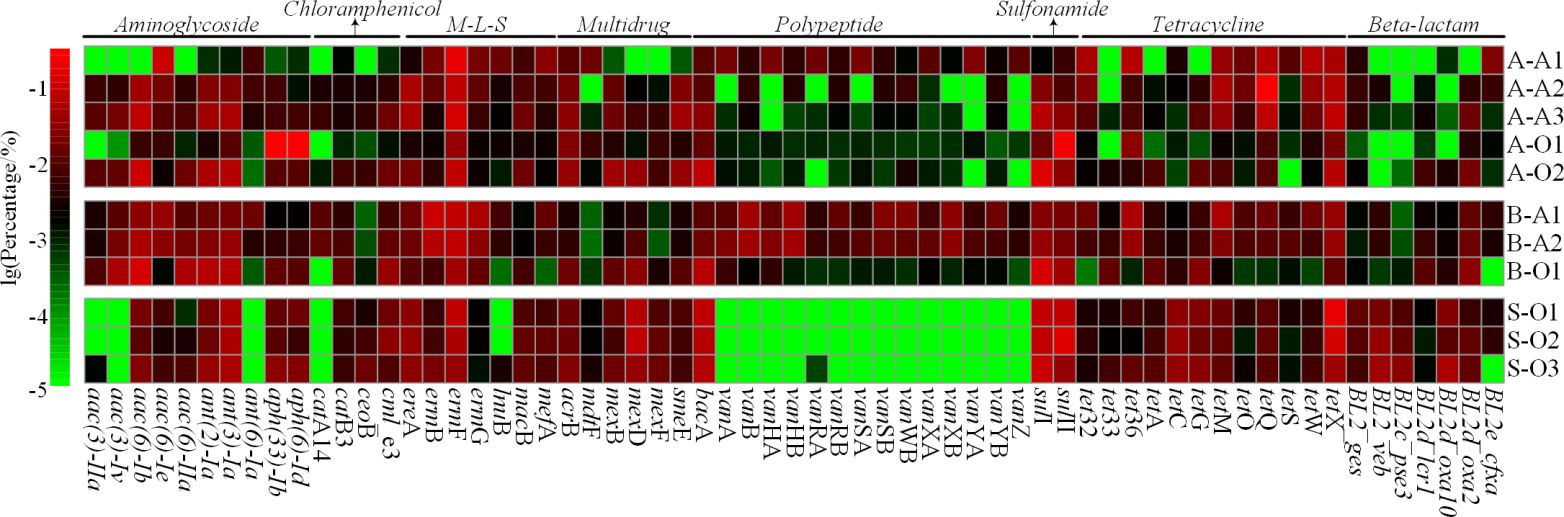
Percentage of the predominant ARG subtypes (≥1% in at least one sludge sample) in PWWTP and STP sludge.

To conduct further analysis, we selected 63 predominant (≥1% in at least one sludge sample) ARG subtypes (accounting for 89.5% to 97.9% of the ARGs in all samples) from 215 ARG subtypes and their percentages were shown in Fig 3. Among the 63 ARG subtypes, 16 subtypes (including 2 aminoglycoside, 2 chloramphenicol, 1 M-L-S, 5 polypeptide, 3 tetracycline and 3 beta-lactam resistance) were only predominant in one sample. While, *erm*F and *tet*X were detected with high percentage (≥1%) in all samples. As mentioned before, the tetracycline resistance genes were detected with very low percentage in aerobic sludge, but *tet*X in this type was persistent in all samples. In addition, the percentage of vancomycin (one kind of polypeptide antibiotics) resistance genes (e.g., *van*A) had 1–2 orders of magnitude lower in aerobic sludge than the anaerobic sludge, especially in STP aerobic sludge. Besides, two subtypes of aminoglycoside, *aph*(33)-Ib (25.86%) and *aph*(6)-Id (29.23%), in A-O1 were obviously higher than other samples.

### PCoA Analysis of ARGs in PWWTP and STP sludge

**Fig 4:**
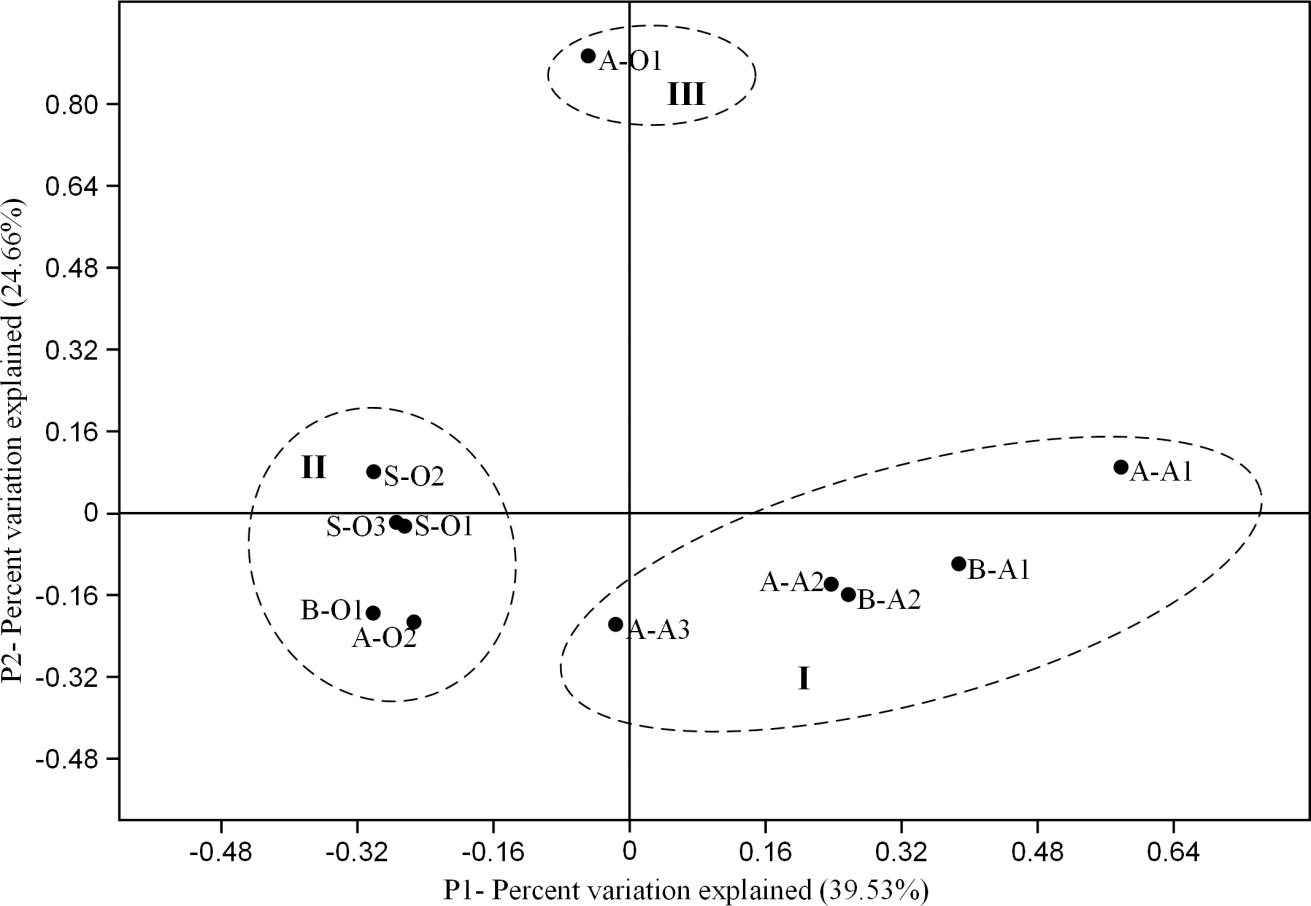
Principal coordinate analysis of 11 PWWTP and STP sludge samples based on the percentages of ARG subtypes.

PCoA (Fig 4) was conducted based on the percentage of ARG subtypes in PWWTP and STP sludge. The PCoA result revealed that the ARG subtypes in the 11 samples could be clustered into three groups: (1) Group I contained all PWWTP anaerobic sludge samples; (2) Group II contained all STP aerobic sludge samples and two PWWTP aerobic sludge samples (A-O2 and B-O1); (3) Group III only contained the PWWTP aerobic sludge sample A-O1. This implies that the dissolved oxygen (DO) might be one of the important factors affecting on the distribution of ARG subtypes in wastewater treatment systems. Moreover, it also confirmed the results mentioned before that the dominated ARG subtypes in PWWTP and STP aerobic sludge were similar.

### Correlations between ARGs and Bacterial Community

**Fig 5:**
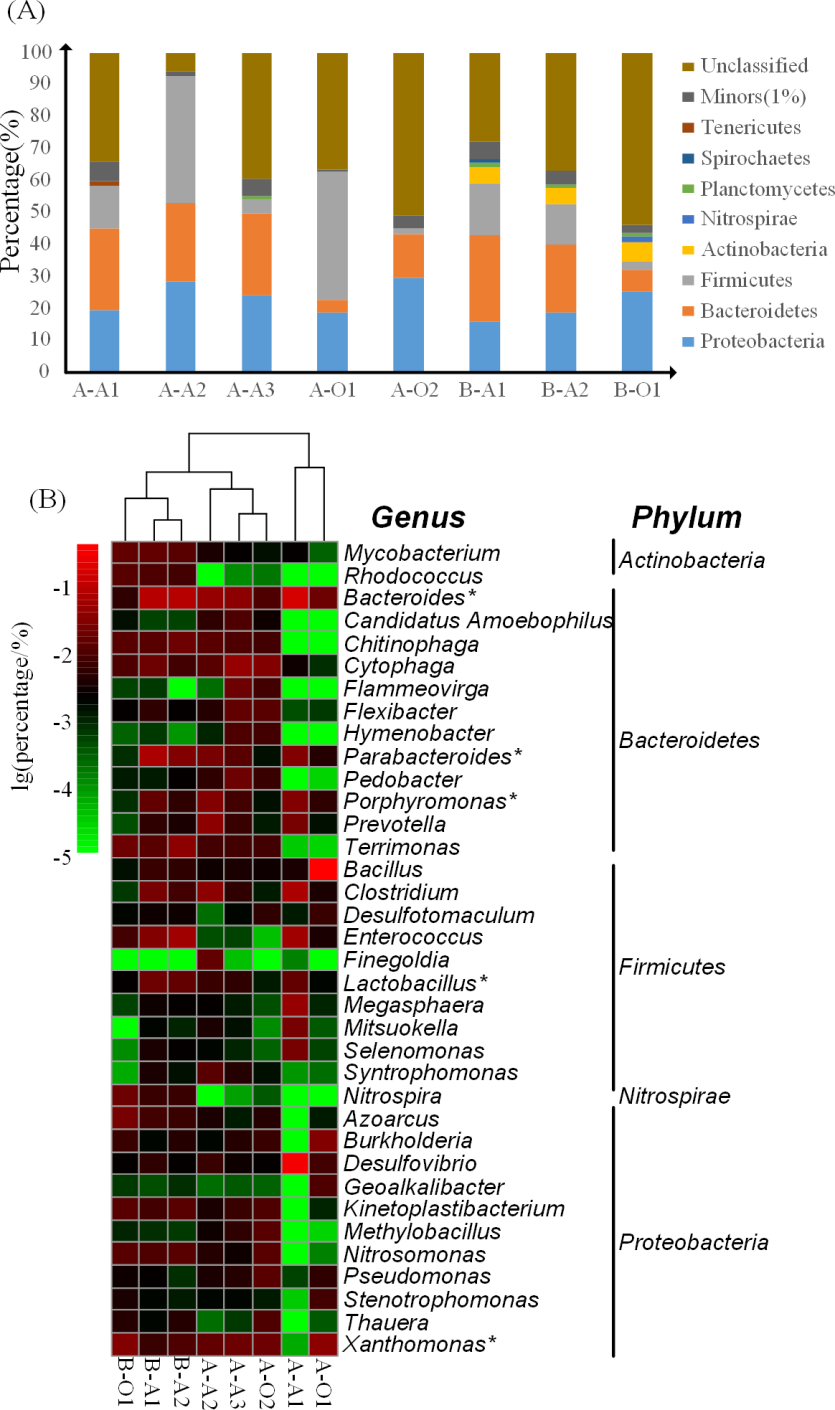
Bacterial community in the PWWTP sludge samples. (A) Percentages of different phyla in the PWWTP sludge. “Others (1%)” refers to the phyla with abundance <1% in all samples. (B) Percentages of top 10 genera in the PWWTP sludge samples. The asterisks indicate the significant difference with *p*-value<0.05 between anaerobic and aerobic sludge samples.

To better understand the dissemination and fate of ARGs in the wastewater treatment systems, we examined the bacterial community in all PWWTP sludge and investigated the correlations between ARGs and bacterial community. The bacterial community of the PWWTP sludge at phylum level was shown in Fig 5A, *Proteobacteria*, *Firmicutes* and *Bacteroidetes* were the three predominant phyla in the eight samples. In general, except for A-O1, the percentage of *Proteobacteria* in the anaerobic sludge (average 21.05%) was lower than its in aerobic sludge (average 27.22%). On the contrary, *Firmicutes* and *Bacteroidetes* were more abundant in anaerobic sludge (average 17.31% and 24.86%, respectively) than those in aerobic sludge (average 2.11% and 8.76%, respectively). The percentages of *Proteobacteria*, *Firmicutes* and *Bacteroidetes* in A-O1 accounted for 18.39%, 40.44% and 3.90%, respectively. The percentages of the bacterial genera in the PWWTP sludge were shown in Fig 5B. To simplify the results, we selected the top 10 abundant genera in each sample (In total, 36 abundant genera were obtained from the 8 samples) for comparison. Among the 36 genera, the percentages of five genera (including *Bacteroides*, *Lactobacillus*, *Parabacteroides*, *Porphyromonas*, *Xanthomonas*) were significantly different between anaerobic sludge and aerobic sludge (*p*<0.05). Besides, the percentage of *Bacillus* in A-O1 (37.3%) was obviously higher than those in other samples.

**Fig 6:**
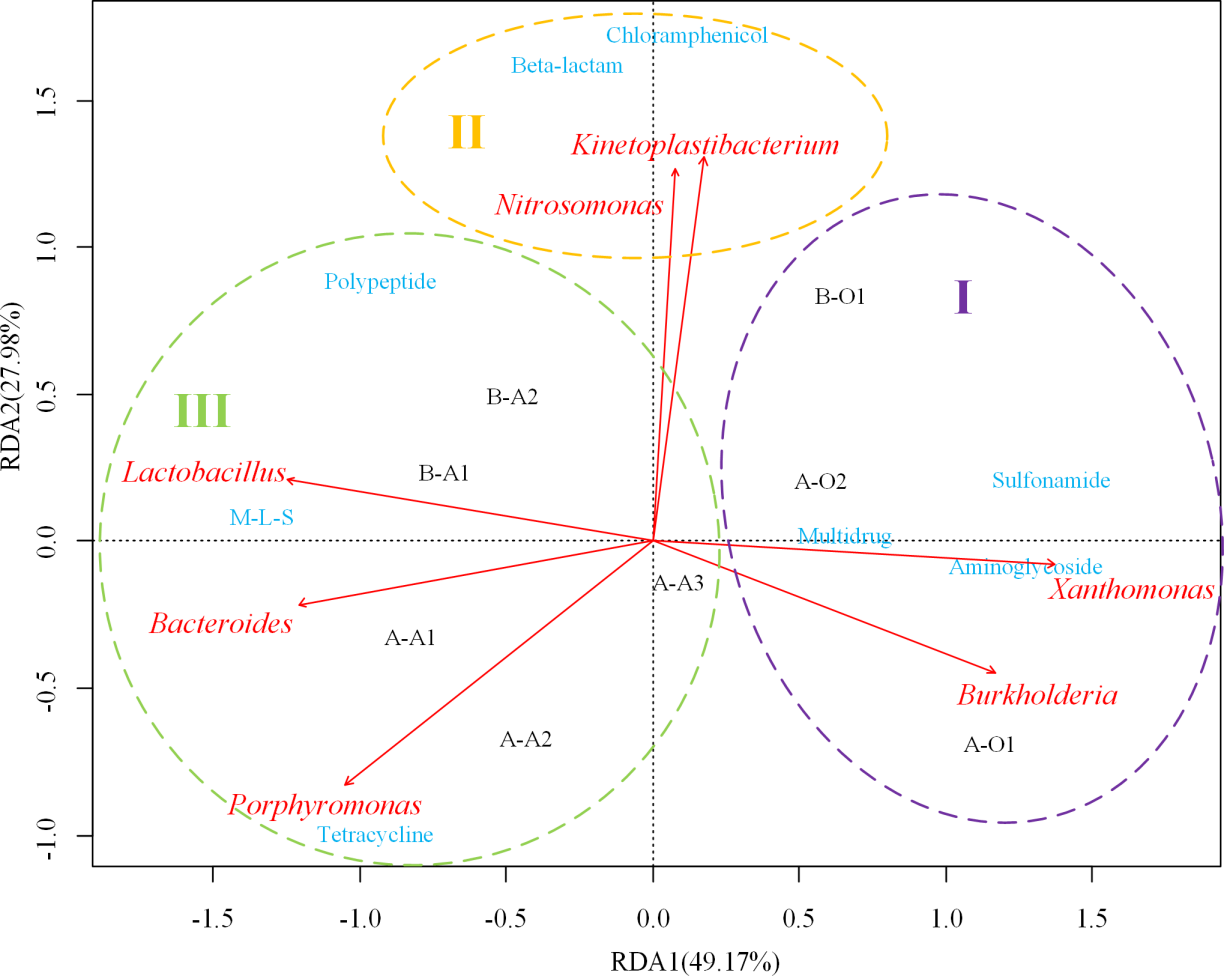
Redundancy analysis of the correlation between the percentages of 36 dominant genera with the percentages of eight ARG types in PWWTP sludge. Arrows represent the seven genera significantly correlated with ARGs distribution (*p*<0.05).

The correlation of bacterial community and ARG types was examined through RDA based on the percentages of the 36 abundant genera and percentages of the 8 ARG types (except others). As shown in Fig 6, among the 36 genera, 7 genera (*Xanthomonas*, *Burkholderia*, *Porphyromonas*, *Bacteroides*, *Lactobacillus*, *Nitrosomonas* and *Kinetoplastibacterium*) were significantly correlated with the ARG types (*p*<0.05), indicating that these genera possibly played important roles in shaping the ARG profiles in the sludge samples. Moreover, the ARG types could be grouped into three clusters according to the RDA results. Cluster I, which included aminoglycoside, sulfonamide and multidrug resistance genes, was positively correlated with *Xanthomonas*, and *Burkholderia*. Cluster II, which included M-L-S, polypeptide and tetracycline resistance genes, was positively correlated with *Porphyromonas*, *Bacteroides* and *Lactobacillus*. Cluster III, which included chloramphenicol and beta-lactam resistance genes, was positively correlated with *Nitrosomonas*, *Kinetoplastibacterium*. In addition, the samples could be grouped into two clusters, one cluster contained all aerobic samples and the other contained all anaerobic samples.

To better understand the correlation, the association between the percentage of predominant ARG subtypes and the seven genera identified in Fig 6 were further analyzed. As shown in S5 Fig, *Burkholderia* was significantly positively correlated with two types of aminoglycoside resistance genes and one type of sulfonamide resistance genes. *Xanthomonas* was significantly positively correlated with one type of sulfonamide resistance genes. *Bacteroides* was significantly positively correlated with three types of M-L-S resistance genes, five types of tetracycline resistance genes and seven types of polypeptide resistance genes. *Lactobacillus* was significantly positively correlated with most of the polypeptide resistance genes (vancomycin resistance genes), two types of tetracycline resistance genes and three types of M-L-S resistance genes. *Porphyromonas* was significantly positively correlated with one type of M-L-S resistance genes and five types of tetracycline resistance genes. Two types of beta-lactam resistance genes and two types of chloramphenicol resistance genes were significantly positively correlated with *Nitrosomonas*, *Kinetoplastibacterium* (*r*>0.5, *p*<0.05).

### Correlations between ARGs and MGEs

In this study, we also examined the MGEs in all PWWTP sludge samples and obvious correlations were observed between ARGs and MGEs. Table 1 summarized the abundance and diversity of MGEs (including integrons, ISs, and plasmids) in all PWWTP sludge samples. The highest abundance of total MGEs was observed in sample B-O1 (6347ppm) and the lowest abundance of total MGEs was observed in sample A-A2 (728ppm). And, the highest and lowest diversity of total MGEs were observed in sample B-A2 (756 types) and sample A-A2 (329 types), respectively. Plasmids were the main MGEs in the PWWTP sludge (>80%) and the plasmid abundance in the aerobic sludge was two times higher than that in the anaerobic sludge (S6 Fig). Furthermore, we summarized the correlation coefficients of the abundance and diversity of ARGs, integrons, ISs, and plasmids in S2 Table. The diversity of ARGs was significantly positively correlated with the diversity of MGEs (*r*>0.5, *p*<0.05). And, the total abundance of ARGs was significant positively correlated with the abundance of MGEs (*r*>0.5, *p*<0.05) (S7 Fig). Finally, as shown in S8 Fig, the abundances of aminoglycoside and sulfonamide resistance genes were significantly correlated with the abundance of plasmids.

**Table 1:** The abundance and diversity of MGEs in PWWTP sludge.

Samples	Insertion sequences		Integrons		Plasmids		Total MGEs^a^	
	Abundance (ppm)^b^	Diversity^c^	Abundance (ppm)	Diversity	Abundance (ppm)	Diversity	Abundance (ppm)	Diversity
A-A1	270	92	2	-	1349	391	1621	483
A-A2	130	86	13	-	585	243	728	329
A-A3	65	147	18	-	761	347	845	494
A-O1	1918	136	43	-	3973	334	5933	470
A-O2	133	132	41	-	1825	290	1999	422
B-A1	548	171	47	-	1706	475	2301	646
B-A2	327	192	84	-	4885	564	5296	756
B-O1	138	154	155	-	6054	448	6347	602

Total MGEs ^a^ = integrons + insertion sequences + plasmids

Abundance ^b^: the portion of MGEs in total metagenomic sequences.

Diversity ^c^: the number of the annotated MGE types.

## Discussion

In this study, metagenomic analysis demonstrated that the abundance of total ARGs in PWWTP sludge was higher than that in STP sludge reported in other studies [34, 35]. Similar results were found by Liu et al. [36] that more abundant tetracycline resistance genes presented in an oxytetracycline wastewater treatment system than those reported in sewage treatment systems. In addition, Liu et al. [19] also reported that the M-L-S resistance genes in spiramycin production wastewater treatment were 2.5 orders of magnitude higher than those in the sewage and inosine wastewater treatment systems. The abundance of total ARGs in the PWWTPs of this study was also obviously higher than those in other environment samples, such as ocean sediments (0.3–7.0ppm) [23], manures and soils (less than 1.0ppm) [21]. To our knowledge, vancomycin resistance genes were rarely detected in the environmental samples [23, 35] at such a high level as in our study. This is probably because vancomycin was the main product of the two PWWTPs (S1 Table). Vancomycin is indicated for treatment of life-threatening infections unresponsive to other antibiotics and it is one of the most important medications needed in a basic health system and was listed in the World Health Organization's List of Essential Medicines (http://www.who.int/medicines/publications/essentialmedicines/en/). The dissemination of vancomycin resistance genes could potentially increase the vancomycin resistance of pathogens and pose serious health risks to humans. Many studies have confirmed that antibiotics can obviously accelerate the accumulation of ARGs and some types of antibiotics could persist for a long time in sludge [8, 37, 38]. Therefore, excess sludge generated during antibiotics production wastewater treatment should be disposed carefully to control the spread of ARGs in the environment.

Consistent with the results of this study, previous studies also showed that tetracycline resistance genes could be frequently detected in WWTPs. Zhang and Zhang [39] found that the tetracycline resistance genes occurred in 15 WWTPs from different locations around the world and Yang et al. [34] found that tetracycline resistance genes were the most abundant ARGs in the activated sludge samples. The tetracycline resistance mechanisms included tetracycline efflux, ribosome protection, tetracycline modification and tetracycline inactivation [40]. In this study, we found that the tetracycline inactivation gene (*tet*X) was predominant in all sludge samples. The reason could be that (1) tetracycline was one of the most commonly used antibiotics for humans and veterinary medicine; (2) tetracycline was significantly adsorbed by activated sludge [37]. A previous study indicated that *tet*X gene was linked to *erm*F because the *erm*F clones (*E*. *coli*) were found to confer tetracycline resistance when grown aerobically [41]. This may be the reason why *erm*F gene was also predominant in all sludge although its corresponding antibiotic was not used as much as tetracycline.

As one of the early studies to compare the distribution of ARGs between PWWTP sludge and STP sludge, our results showed that the distribution of ARGs in PWWTP aerobic sludge was more similar to that in STP aerobic sludge than that in PWWTP anaerobic sludge (PCoA in Fig 4). This suggested that the concentration of DO probably have more impacts than the influent composition on the distribution of ARGs. Besides, it was noticed that the sample A-O1 was separated from other samples in Fig 4 due to the distinct distribution of ARGs in this sample (the resistance genes of *aph*(33)-Ib and *aph*(6)-Id accounted for over 50% of the total abundance of ARGs). This pattern could be attributed to the distinct bacterial community [42, 43]—the abundance of *Bacillus* (37.3%) in A-O1 was obviously higher than those in other samples. Although there was no evidence showing that *Bacillus* carry these two genes, it was reported in some studies [44, 45] that *Bacillus* have aminoglycoside resistance. In addition, according to ARDB, the similar aminoglycoside resistance genes (e.g., *aph*(3)-Ia and *aph*(3)-IIIa) were frequently found in bacteria affiliated to this genus. MGEs may be another factor shaping the distribution of ARGs in sample A-O1 [46]. Aminoglycoside resistance genes were often detected in the plasmids [47], and the abundance of MGEs in A-O1 was 3–8 times higher than those in other samples of PWWTP A.

It was known that different kinds of antibiotics display different bactericidal activity for specific group of bacteria. For instance, Aminoglycoside and sulfonamide displays bactericidal activity against Gram-negative aerobes [48, 49]. Polypeptide and Macrolide (M-L-S) have effects on Gram-positive bacteria [50, 51]. Chloramphenicol and beta-lactam were used to treat infections caused by both Gram-positive and Gram-negative bacteria [52, 53]. Tetracycline would highly enriched *Bacteroidetes* in the sludge [54]. It was also known that the selective pressure from antibiotics could accelerate the ARGs transmission in the sensitive bacteria [8]. Therefore, bacteria could develop resistance genes to resist their specific antibiotics. Thus, the aminoglycoside and sulfonamide resistance genes may frequently occur in Gram-negative bacteria. And, polypeptide and M-L-S resistance genes may frequently occur in Gram-positive bacteria. Most *Proteobacteria* and *Bacteroidetes* are Gram-negative bacteria and most *Firmicutes* are Gram-positive bacteria [55]. Based on the above information, it can be inferred that distribution of ARGs may change with the alteration of bacterial community. Previous studies [25, 56–57] have shown that DO could impact on the microbial community structure and the dominant bacterial population might change from *Proteobacteria* to *Firmicutes* and *Bacteroidetes* with the decrease of DO. This implies that the shifts of microbial community affected by DO would affect the distribution of ARGs. Besides of DO, temperature could also impact the microbial community structure [58]. In this study, the temperature in the aerobic and anaerobic bioreactors of PWWTPs were similar (30±2^°^C). Further studies are needed to investigate whether temperate could also impact the ARGs profiles through affecting microbial community in bioreactors.

In this study, the percentages of *Xanthomonas*, *Bacteroides*, *Lactobacillus*, *Porphyromonas* genera were significantly different between anaerobic sludge and aerobic sludge and these five genera were significantly correlated with the distribution of ARG types. The subtypes of the correlated ARG types have been frequently detected in those genera. For example, *tet*Q, *erm*F and vancomycin resistance genes were detected in *Porphyromonas*, *Bacteroides* and *Lactobacillus* [59–62]. According to ARDB, species in *Xanthomonas* were aerobic Gram-negative bacteria and were found to carry *aph*(33)-Ib and *aph*(6)-Id. As another aerobic Gram-negative bacterial genus, *Burkholderia* demonstrated a high-level of antibiotic resistance, including aminoglycoside, macrolide and resistance-nodulation-cell division (RND) transportation system (most of multidrug resistance genes belong to RND transportation system) [63]. These results confirmed that the distribution of ARGs could be affected by the microbial community.

## Conclusions

In this study, ARGs in two PWWTPs with aerobic and anaerobic systems and three STP aerobic systems were investigated. The results showed that the diversity and abundance of ARGs in PWWTPs were higher than those in STPs, suggesting that PWWTP sludge, as an important source of ARG pollution, need to be paid more attention to. The distribution of ARGs in PWWTP aerobic sludge was more similar to those in STP aerobic sludge than those in PWWTP anaerobic sludge. Tetracycline, M-L-S and polypeptide (especially vancomycin) resistance genes could be accumulated in anaerobic treatment system. While, aminoglycoside, sulfonamide and multidrug resistance genes were abundantly present in aerobic treatment system. Microbial community between the aerobic and anaerobic sludge were different and significant correlations were observed between the ARGs and microbial community. The abundance and diversity of MGEs were also positively correlated with the ARGs and the ARGs in the plasmids were detected more frequently in aerobic sludge than those in anaerobic sludge. These results imply that the shifts of microbial community and MGEs as controlled by DO may affect the distribution of ARGs in PWWTP bioreactors.

## Supporting Information

S1 Fig The flow chart of the treatment processes in the three STPs. The black points in oxidation ditch represent the sampling sites.
(DOCX)

S2 Fig The diversity of ARGs in the PWWTPs and STPs sludge. (DOCX)

S3 Fig Average percentages of different ARG types in PWWTP anaerobic sludge (P-A), PWWTPs aerobic sludge (P-O) and STP aerobic sludge (S-O). (DOCX)

S4 Fig Average percentages of different resistance mechanisms in PWWTPs anaerobic sludge (P-A), PWWTP aerobic sludge (P-O) and STP aerobic sludge (S-O). (DOCX)

S5 Fig Heatmap showing the Pearson correlation coefficient between the seven genera and the predominant ARG subtypes (at least ≥1% in one sludge sample) in the PWWTPs sludge. Correlation coefficient between the genus and ARG subtypes at r>0.5&p<0.05 was marked “★”. Correlation coefficient between the genus and ARG subtypes at r>0.5&p≥0.05 was marked “☆”.
(DOCX)

S6 Fig Average abundances of different MGEs in PWWTP anaerobic sludge (P-A) and aerobic sludge (P-O). (DOCX)

S7 Fig Correlations of the abundance (A) and diversity (B) between ARGs and MGEs in the PWWTPs sludge.
(DOCX)

S8 Fig Correlations between the abundance of MGEs/Plasmids and two specific ARGs (aminoglycoside resistance genes (A) and sulfonamide resistance genes (B)) in the PWWTPs sludge.
(DOCX)

S1 File Diversity and abundance of shared ARG subtypes between PWWTPs aerobic sludge (P-O) and STPs aerobic sludge (S-O). (A) Numbers of shared ARG subtypes by PWWTPs aerobic sludge and STPs aerobic sludge. (B) Percentages of the shared ARG subtypes in total ARGs.
(DOCX)

S1 Table Characteristics of two PWWTPs and three STPs. HA, Hydrolytic Acidification; CASS, Cyclic Activated Sludge System; A/O, Anaerobic/Aerobic; UBF, Up-flow Blanket Filter; A/O*, Anoxic/Aerobic; A/A/O, Anaerobic/Anoxic/Aerobic; OD, oxidation ditch.
(DOCX)

S2 Table The correlation coefficients between ARGs and MGEs in the PWWTPs sludge. MGEs = integron + insertion sequence + plasmid ^A^: Abundance ^D^: Diversity ‘*’: indicates significant correlation (*p*<0.05).
(DOCX)
