# Supplementary material for: High Levels of Antibiotic Resistance Genes and Their Correlations with Bacterial Community and Mobile Genetic Elements in Pharmaceutical Wastewater Treatment Bioreactors
Source: PLoS One. 2016 Jun 13;11(6):e0156854. doi: 10.1371/journal.pone.0156854 (PMC4905627; doi:10.1371/journal.pone.0156854)

**S1 Fig. The flow chart of the treatment processes in the three STPs.** The black points in oxidation ditch represent the sampling sites.


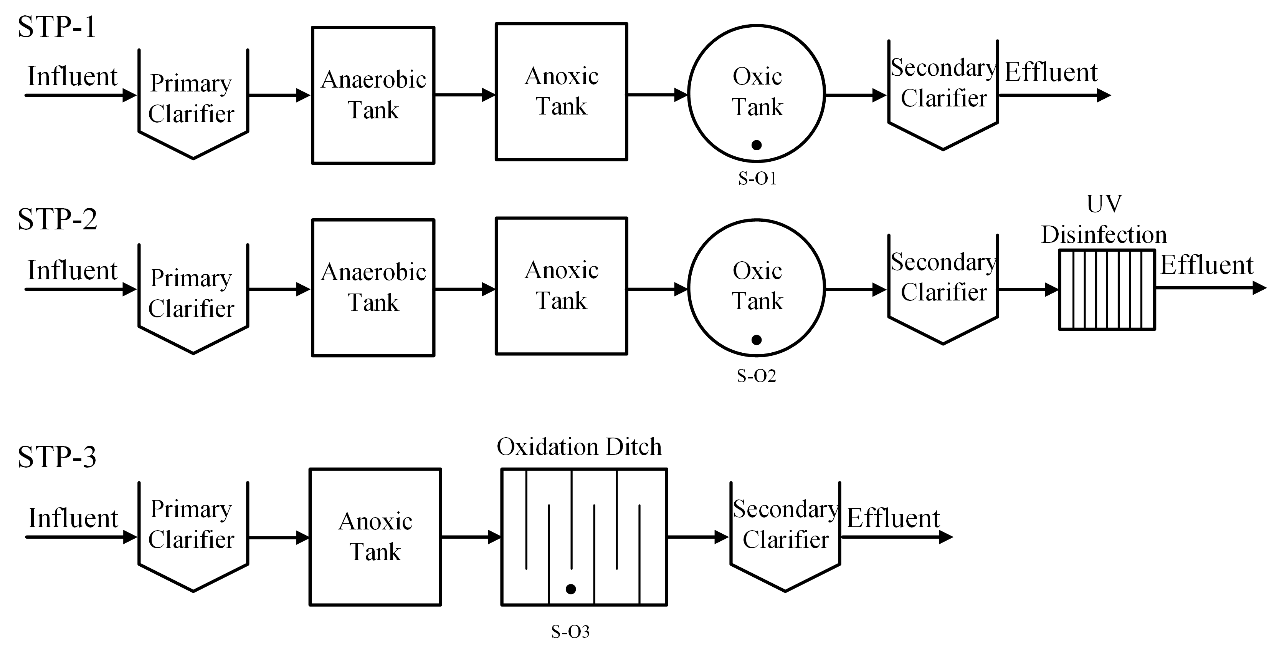

Supplement: S1 Fig — The black points in oxidation ditch represent the sampling sites. (DOCX) [file pone.0156854.s001.docx]
