# Supplementary figures and images for: High Levels of Antibiotic Resistance Genes and Their Correlations with Bacterial Community and Mobile Genetic Elements in Pharmaceutical Wastewater Treatment Bioreactors
Source: PLoS One. 2016 Jun 13;11(6):e0156854. doi: 10.1371/journal.pone.0156854 (PMC4905627; doi:10.1371/journal.pone.0156854)

**S2 Fig. The diversity of ARGs in the PWWTPs and STPs sludge.**


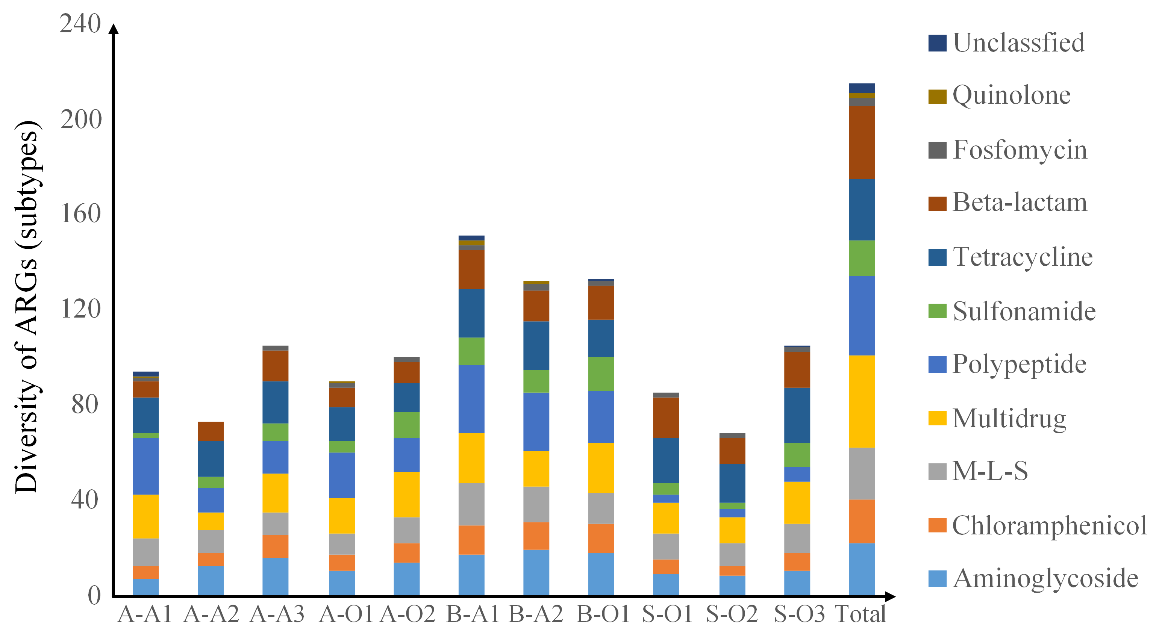

Supplement: S2 Fig — (DOCX) [file pone.0156854.s002.docx]

**S7 Fig. Average abundances of different MGEs in PWWTP anaerobic sludge (P-A) and aerobic sludge (P-O).**


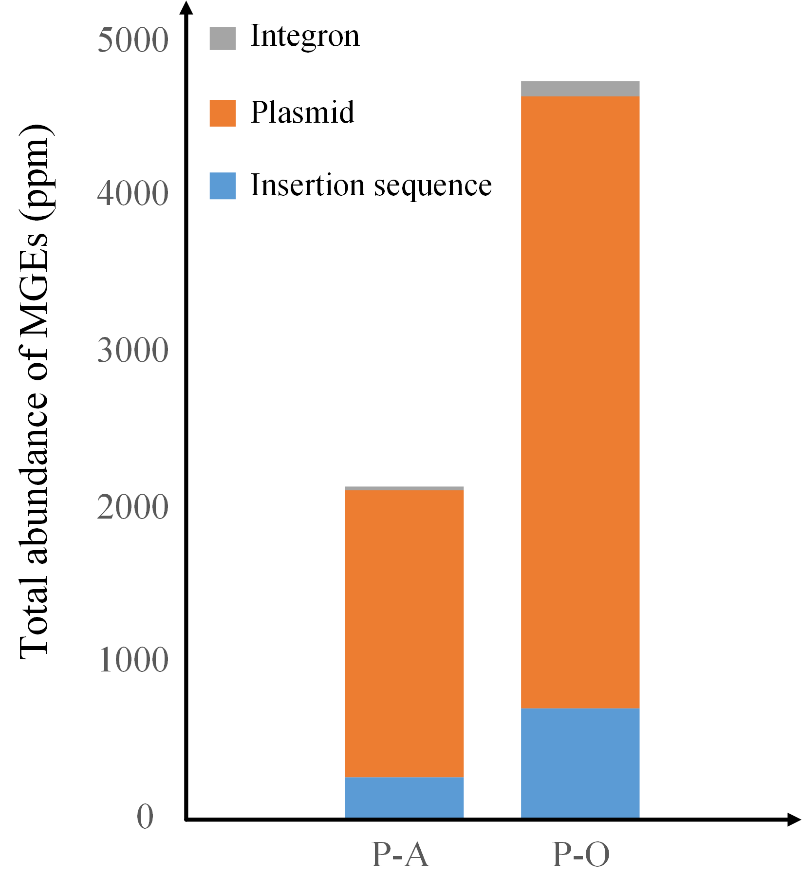

Supplement: S6 Fig — (DOCX) [file pone.0156854.s006.docx]

**S8 Fig. Correlations of the abundance (A) and diversity (B) between ARGs and MGEs in the PWWTPs sludge.**


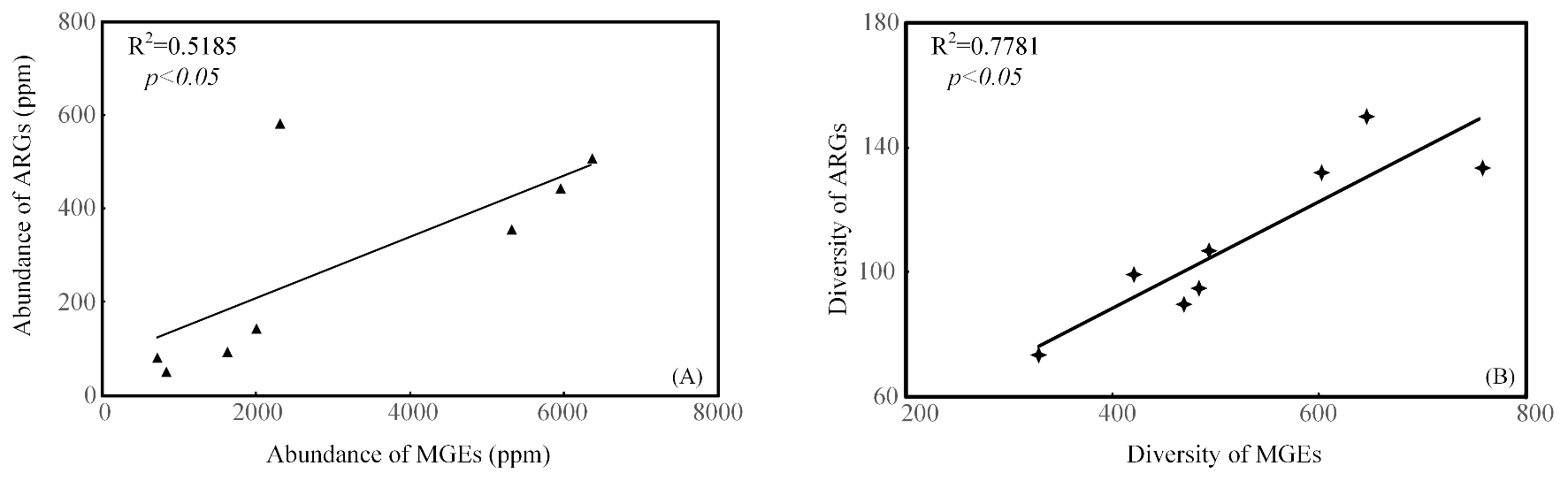

Supplement: S7 Fig — Correlations of the abundance (A) and diversity (B) between ARGs and MGEs in the PWWTPs sludge. (DOCX) [file pone.0156854.s007.docx]
