# Supplementary material for: High Levels of Antibiotic Resistance Genes and Their Correlations with Bacterial Community and Mobile Genetic Elements in Pharmaceutical Wastewater Treatment Bioreactors
Source: PLoS One. 2016 Jun 13;11(6):e0156854. doi: 10.1371/journal.pone.0156854 (PMC4905627; doi:10.1371/journal.pone.0156854)

**S4 Fig. Average percentages of different ARG types in PWWTP anaerobic sludge (P-A), PWWTPs aerobic sludge (P-O) and STP aerobic sludge (S-O).**


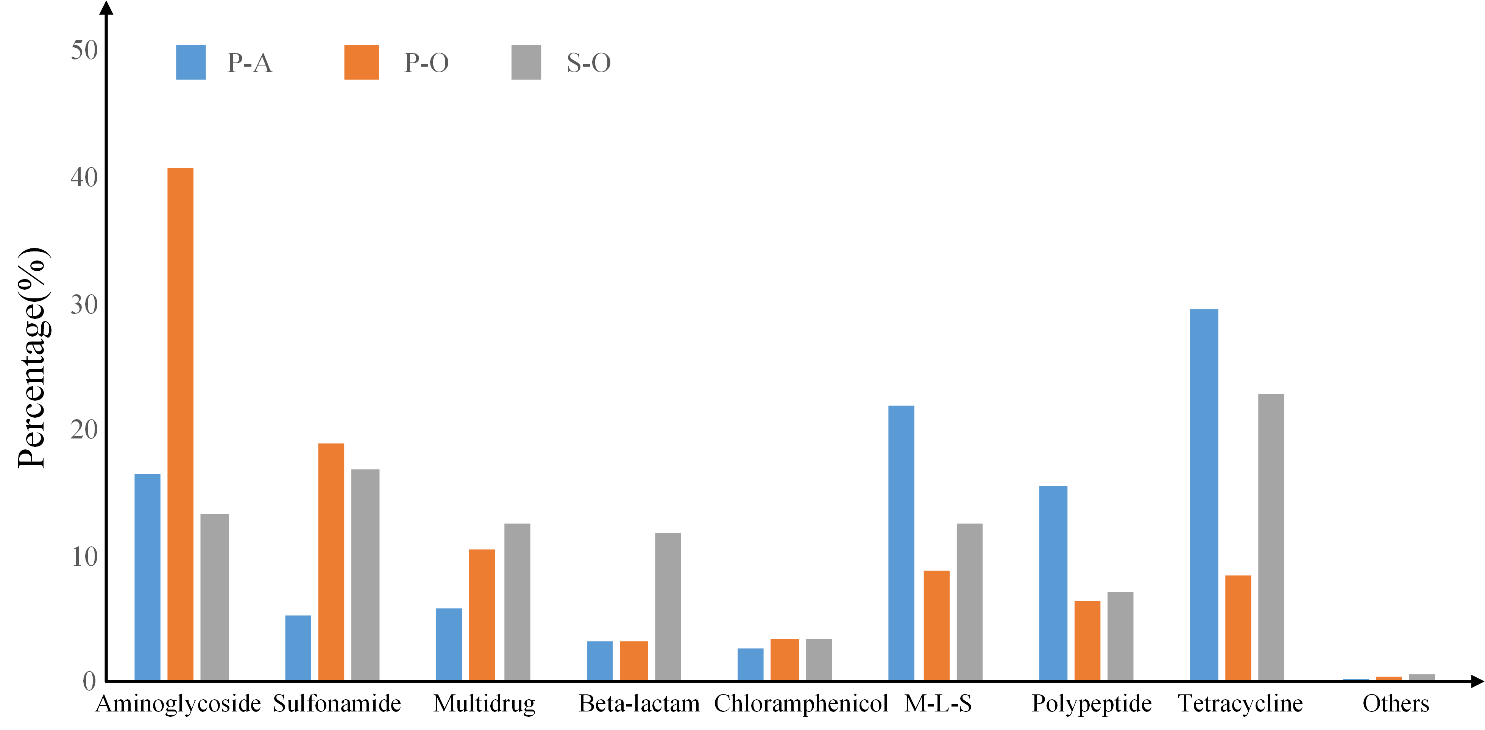

Supplement: S3 Fig — (DOCX) [file pone.0156854.s003.docx]
