# Supplementary material for: High Levels of Antibiotic Resistance Genes and Their Correlations with Bacterial Community and Mobile Genetic Elements in Pharmaceutical Wastewater Treatment Bioreactors
Source: PLoS One. 2016 Jun 13;11(6):e0156854. doi: 10.1371/journal.pone.0156854 (PMC4905627; doi:10.1371/journal.pone.0156854)

**S5 Fig. Average percentages of different resistance mechanisms in PWWTPs anaerobic sludge (P-A), PWWTP aerobic sludge (P-O) and STP aerobic sludge (S-O).**


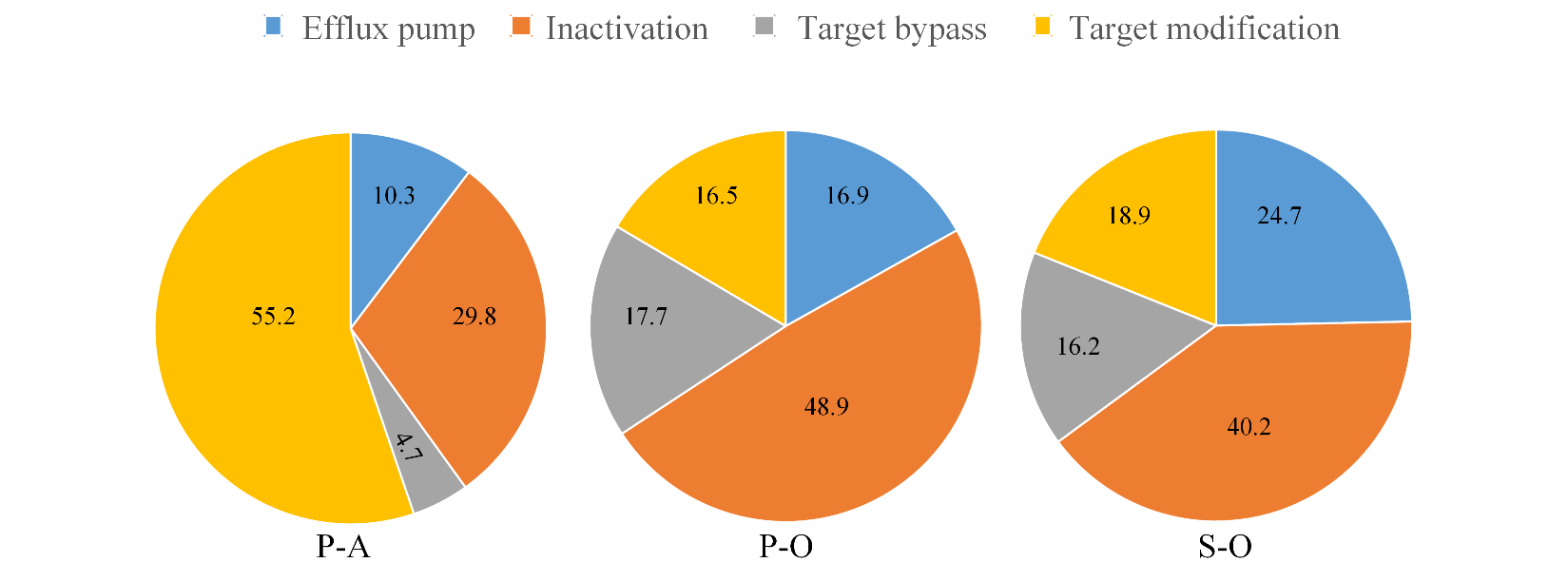

Supplement: S4 Fig — (DOCX) [file pone.0156854.s004.docx]
