# Supplementary material for: High Levels of Antibiotic Resistance Genes and Their Correlations with Bacterial Community and Mobile Genetic Elements in Pharmaceutical Wastewater Treatment Bioreactors
Source: PLoS One. 2016 Jun 13;11(6):e0156854. doi: 10.1371/journal.pone.0156854 (PMC4905627; doi:10.1371/journal.pone.0156854)

**S6 Fig. Heatmap showing the Pearson correlation coefficient between the seven genera and the predominant ARG subtypes (at least ≥1% in one sludge sample) in the PWWTPs sludge.** Correlation coefficient between the genus and ARG subtypes at r>0.5&p<0.05 was marked “★”. Correlation coefficient between the genus and ARG subtypes at r>0.5&p≥0.05 was marked “☆”.


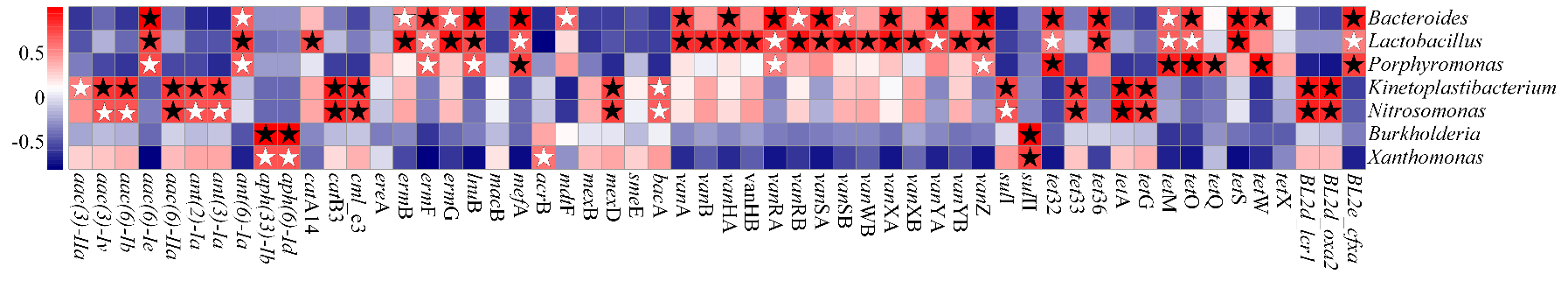

Supplement: S5 Fig — Correlation coefficient between the genus and ARG subtypes at r>0.5&p<0.05 was marked “★”. Correlation coefficient between the genus and ARG subtypes at r>0.5&p≥0.05 was marked “☆”. (DOCX) [file pone.0156854.s005.docx]
