# Supplementary material for: High Levels of Antibiotic Resistance Genes and Their Correlations with Bacterial Community and Mobile Genetic Elements in Pharmaceutical Wastewater Treatment Bioreactors
Source: PLoS One. 2016 Jun 13;11(6):e0156854. doi: 10.1371/journal.pone.0156854 (PMC4905627; doi:10.1371/journal.pone.0156854)

**S9 Fig. Correlations between the abundance of MGEs/Plasmids and two specific ARGs (aminoglycoside resistance genes (A) and sulfonamide** **resistance genes (B) ) in the PWWTPs sludge.**


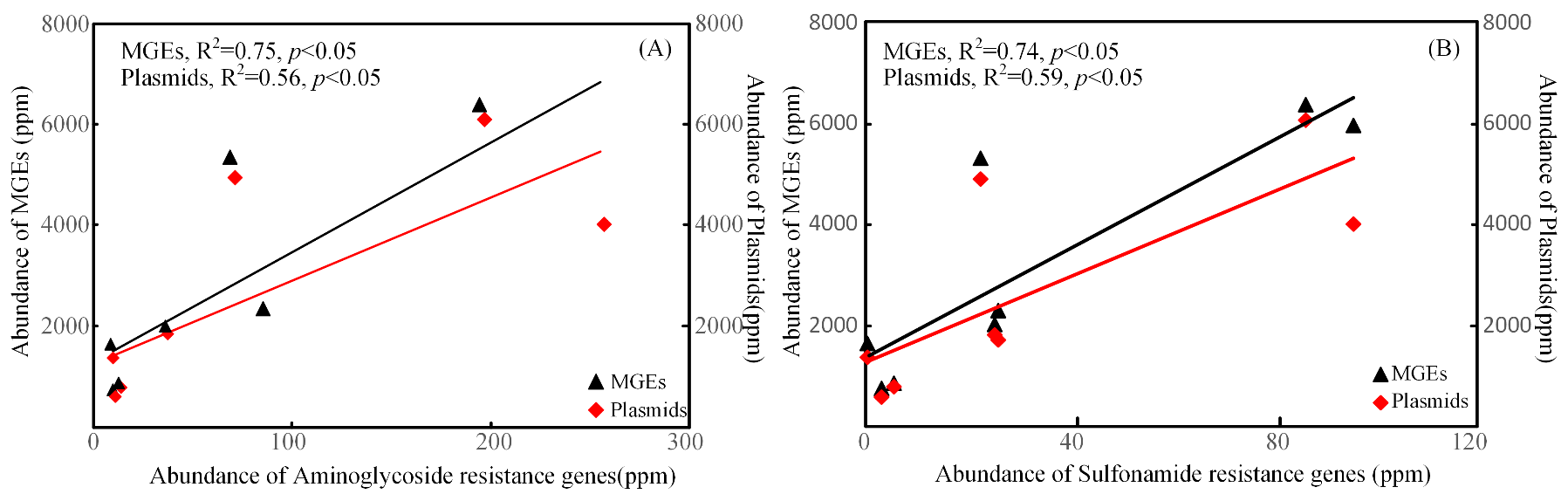

Supplement: S8 Fig — Correlations between the abundance of MGEs/Plasmids and two specific ARGs (aminoglycoside resistance genes (A) and sulfonamide resistance genes (B)) in the PWWTPs sludge. (DOCX) [file pone.0156854.s008.docx]
