# Supplementary material for: High Levels of Antibiotic Resistance Genes and Their Correlations with Bacterial Community and Mobile Genetic Elements in Pharmaceutical Wastewater Treatment Bioreactors
Source: PLoS One. 2016 Jun 13;11(6):e0156854. doi: 10.1371/journal.pone.0156854 (PMC4905627; doi:10.1371/journal.pone.0156854)

**S3. Diversity and abundance of shared ARG subtypes between PWWTPs aerobic sludge (P-O) and STPs aerobic sludge (S-O).** (A) Numbers of shared ARG subtypes by PWWTPs aerobic sludge and STPs aerobic sludge. (B) Percentages of the shared ARG subtypes in total ARGs.


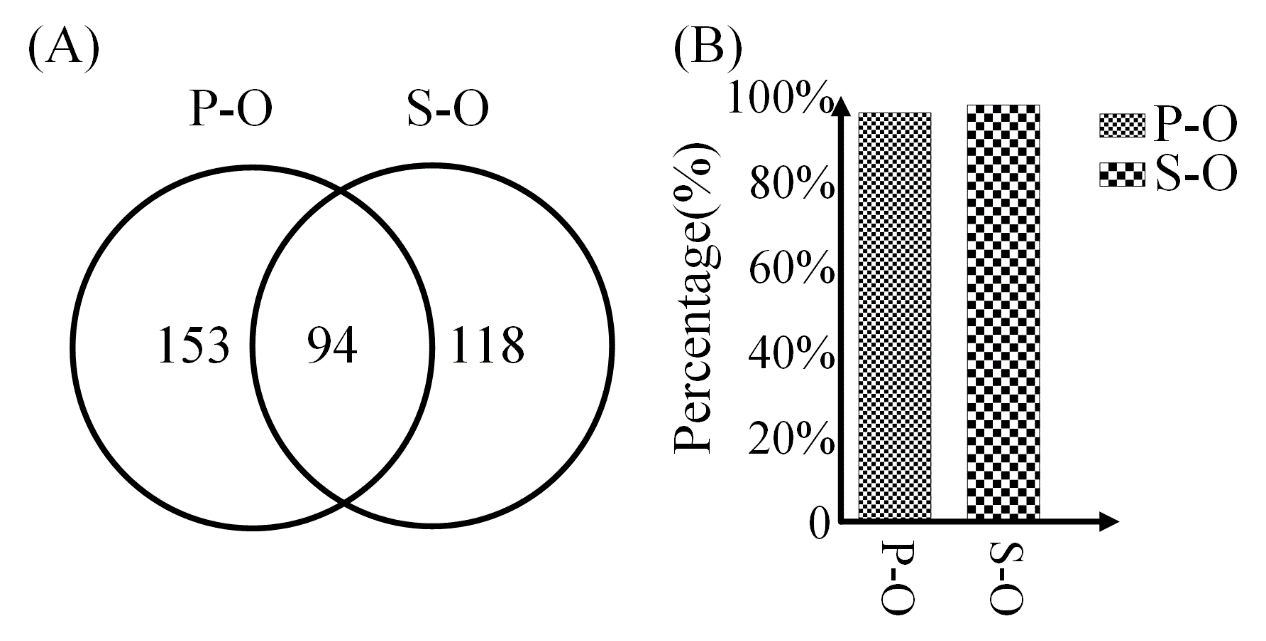

Supplement: S1 File — (A) Numbers of shared ARG subtypes by PWWTPs aerobic sludge and STPs aerobic sludge. (B) Percentages of the shared ARG subtypes in total ARGs. (DOCX) [file pone.0156854.s009.docx]
