# Supplementary material for: High Levels of Antibiotic Resistance Genes and Their Correlations with Bacterial Community and Mobile Genetic Elements in Pharmaceutical Wastewater Treatment Bioreactors
Source: PLoS One. 2016 Jun 13;11(6):e0156854. doi: 10.1371/journal.pone.0156854 (PMC4905627; doi:10.1371/journal.pone.0156854)
Supplement: S1 Table — HA, Hydrolytic Acidification; CASS, Cyclic Activated Sludge System; A/O, Anaerobic/Aerobic; UBF, Up-flow Blanket Filter; A/O*, Anoxic/Aerobic; A/A/O, Anaerobic/Anoxic/Aerobic; OD, oxidation ditch. (DOCX) [file pone.0156854.s010.docx]

**S1 Table. Characteristics of two PWWTPs and three STPs.**

| Sample ID | CODcr | T-N | NH_4_^+^ | T-P | Cl^-^ | SO_4_^2-^ | WWTP ID | Location | Process | Main Products |
| --- | --- | --- | --- | --- | --- | --- | --- | --- | --- | --- |
|  | (mg/L) | | | | | |  |  |  |  |
| A-A1 | 20200 | 292 | 183 | 20 | 1030 | 189 | PWWTPA | Hangzhou,China | HA&CASS&A/O | Vancomycin,Enramycin,Hygromycin |
| A-A2 | 3640 | 89 | 83 | 17 | 1361 | 198 | PWWTPA | Hangzhou,China | HA&CASS&A/O | Vancomycin,Enramycin,Hygromycin |
| A-A3 | 1120 | 75 | 71 | 10 | 1053 | 77 | PWWTPA | Hangzhou,China | HA&CASS&A/O | Vancomycin,Enramycin,Hygromycin |
| A-O1 | 2893 | 141 | 120 | 15 | 897 | 551 | PWWTPA | Hangzhou,China | HA&CASS&A/O | Vancomycin,Enramycin,Hygromycin |
| A-O2 | 320 | 16 | 4 | 6 | 828 | 425 | PWWTPA | Hangzhou,China | HA&CASS&A/O | Vancomycin,Enramycin,Hygromycin |
| B-A1 | 840 | 322 | 319 | 2 | 1530 | 1525 | PWWTPB | Taizhou,China | UBF&A/O* | Vancomycin,Enramycin,Hygromycin |
| B-A2 | 1907 | 334 | 287 | 27 | 1564 | 1680 | PWWTPB | Taizhou,China | UBF&A/O* | Vancomycin,Enramycin,Hygromycin |
| B-O1 | 453 | 88 | 49 | 29 | 1520 | 530 | PWWTPB | Taizhou,China | UBF&A/O* | Vancomycin,Enramycin,Hygromycin |
| S-O1 | NA | NA | NA | NA | NA | NA | STP-1 | Zheng zhou,China | A/A/O | NA |
| S-O2 | NA | NA | NA | NA | NA | NA | STP-2 | Zheng zhou,China | A/A/O | NA |
| S-O3 | NA | NA | NA | NA | NA | NA | STP-3 | Zheng zhou,China | OD | NA |

HA, Hydrolytic Acidification; CASS, Cyclic Activated Sludge System; A/O, Anaerobic/Aerobic; UBF, Up-flow Blanket Filter; A/O*, Anoxic/Aerobic; A/A/O, Anaerobic/Anoxic/Aerobic; OD, oxidation ditch.
