# Supplementary material for: High Levels of Antibiotic Resistance Genes and Their Correlations with Bacterial Community and Mobile Genetic Elements in Pharmaceutical Wastewater Treatment Bioreactors
Source: PLoS One. 2016 Jun 13;11(6):e0156854. doi: 10.1371/journal.pone.0156854 (PMC4905627; doi:10.1371/journal.pone.0156854)
Supplement: S2 Table — MGEs = integron + insertion sequence + plasmid A: Abundance D: Diversity ‘*’: indicates significant correlation (p<0.05). (DOCX) [file pone.0156854.s011.docx]

**S2 Table. The correlation coefficients between ARGs and MGEs in the PWWTPs sludge.**

|  | Insertion sequence^D^ | Plasmid^D^ | MGEs^D^ | Insertion sequence^A^ | Plasmid^A^ | Integron^A^ | MGEs^A^ |
| --- | --- | --- | --- | --- | --- | --- | --- |
| ARG^A^ | 0.64392 | 0.60268 | 0.63938 | 0.45615 | 0.66675 | 0.6561 | 0.72006* |
| ARG^D^ | 0.84284* | 0.84712* | 0.88208* | -0.13605 | 0.46937 | 0.61304 | 0.38939 |

MGEs = integron + insertion sequence + plasmid

^A^: Abundance ^D^: Diversity ‘*’: indicates significant correlation (*p*<0.05)
